# Efficient Recycling Processes for Lithium-Ion Batteries

**DOI:** 10.3390/ma18030613

**Published:** 2025-01-29

**Authors:** Sabyasachi Paul, Pranav Shrotriya

**Affiliations:** Mechanical Engineering, Iowa State University, Ames, IA 50011, USA; ssachi@iastate.edu

**Keywords:** spent Li-ion battery, battery recycling, direct recycling, regeneration, resynthesis

## Abstract

Lithium-ion batteries (LIBs) are an indispensable power source for electric vehicles, portable electronics, and renewable energy storage systems due to their high energy density and long cycle life. However, the exponential growth in production and usage has necessitated highly effective recycling of end-of-life LIBs to recover valuable resources and minimize the environmental impact. Pyrometallurgical and hydrometallurgical processes are the most common recycling methods but pose considerable difficulties. The energy-intensive pyrometallurgical recycling process results in the loss of critical materials such as lithium and suffers from substantial emissions and high costs. Solvent extraction, a hydrometallurgical method, offers energy-efficient recovery for lithium, cobalt, and nickel but requires hazardous chemicals and careful waste management. Direct recycling is an alternative to traditional methods as it preserves the cathode active material (CAM) structure for quicker and cheaper regeneration. It also offers environmental advantages of lower energy intensity and chemical use. Hybrid pathways, combining hydrometallurgical and direct recycling methods, provide a cost-effective, scalable solution for LIB recycling, maximizing material recovery with minimal waste and environmental risk. The success of recycling methods depends on factors such as battery chemistry, the scalability of recovery processes, and the cost-effectiveness of waste material recovery. Though pyrometallurgical and hydrometallurgical processes have secured their position in LIB recycling, research is proceeding toward newer approaches, such as direct and hybrid methods. These alternatives are more efficient both environmentally and in terms of cost with a broader perspective into the future. In this review, we describe the current state of direct recycling as an alternative to traditional pyrometallurgical and hydrometallurgical methods for recuperating these critical materials, particularly lithium. We also highlight some significant advancements that make these objectives possible. As research progresses, direct recycling and its variations hold great potential to reshape the way LIBs are recycled, providing a sustainable pathway for battery material recovery and reuse.

## 1. Introduction

Lithium-ion batteries (LIBs) are becoming essential in the rapidly shifting landscape of technology, powering not just the sleek electric vehicles (EVs) that glide through our streets but also the myriads of portable gadgets that pepper our daily lives. They have become the heart of a greener shift, contributing to reducing environmental pollution and finding the way for sustainable energy consumption. Li-ion batteries (LIBs) are emerging as a powerful alternative to lead–acid batteries, particularly in energy storage applications for consumer electronics, electric vehicles (EVs), and industrial uses. Their adoption is driven by advantages such as longer lifespan and higher power density [1]. Lithium (Li) batteries hold the highest cell potential as Li has the lowest reduction potential of all elements. Although multivalent cations have a greater charge capacity per ion, their mobility is significantly decreased by the extra charge. The rate-limiting element for battery power output is often ionic diffusion in solid electrodes, a significant obstacle to developing alternative chemistries [2].

Initially proposed in the 1970s and commercialized by Sony in 1991, LIBs are now pivotal in the transport sector, with their demand for electric vehicles (EVs) expected to surge, significantly reducing battery storage costs by 2030. LIBs dominate the market for electric vehicles (EVs) because of their high energy and power density, extended lifespan, and significant cost reductions over the past several years [3]. Plug-in hybrid electric vehicles (PHEVs), fuel cell electric vehicles (FCEVs), hybrid electric vehicles (HEVs), and battery electric vehicles (BEVs) are among the different types of EVs that use LIBs. It is expected that there will be 253 million electric vehicles (EVs) on the road by 2030 [4]. The demand for electric vehicle (EV) batteries is expected to increase from 340 GWh (in 2024) to over 3500 GWh (by 2030) [5]. Li-ion batteries have low recycling rates, but simpler chemistry and regulatory requirements ensure lead–acid batteries’ 99% recycling rate [6]. Due to consumers’ strong demand for LIBs, effective end-of-life (EOL) recycling options must be considered to guarantee infrastructure readiness.

**Figure 1:**
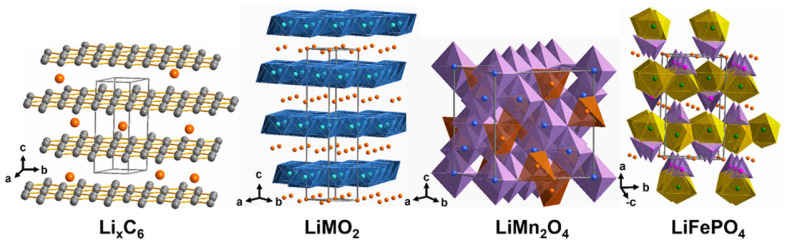
Crystal structures of typical lithium-ion battery electrode materials: Li_x_C_6_ (gray: carbon layers, orange: lithium), LiMO_2_ (blue: MO_6_ octahedra, layered structure), LiMn_2_O_4_ (purple: MnO_6_ octahedra, spinel structure), and LiFePO_4_ (yellow: FeO_6_ octahedra, purple: PO_4_ tetrahedra, olivine structure (reproduced with permission from [7]).

Li-ion batteries consist of a cathode, anode, electrolyte, and separator. The electrode is made of cathode active materials, a carbon conductive additive for electron transport, a polymeric binder, and a current collector. High enough carbon additive levels create a conductive network. PVDF is a standard binder, but eco-friendly alternatives like CMC or SBR are becoming popular. Aluminum is used for the cathode’s current collector due to its resistance to oxidative corrosion, while copper is preferred for the anode to avoid alloying with lithium [7]. Figure 1 depicts the intercalation of lithium within the graphitic structure, with the crystalline structures formed by various cathode active materials that contain entrapped lithium ions post-charging. The stability and performance of the material may be impacted by the alteration in the crystal lattice caused by lithium intercalation. During charge and discharge cycles, the structure of some cathode materials can change due to a phase transition that occurs when the material absorbs or releases lithium ions.

Lithium-ion batteries (LIBs) can be categorized into various varieties based on the available cathode materials, including:

- Lithium cobalt oxide (LiCoO_2_)—LCO;
- Lithium nickel manganese cobalt oxide (LiNiMnCoO_2_)—NMC;
- Lithium Manganese Oxide (LiMn_2_O_4_)—LMO;
- Lithium Nickel Oxide (LiNiO_2_)—LNO;
- Lithium Nickel Cobalt Aluminum Oxide (LiNiCoAlO_2_)—NCA;
- Lithium-ion Phosphate (LiFePO_4_)—LFP;
- Lithium Titanate (Li_4_Ti_5_O_12_)—LTO.

The complexity of Li-ion batteries, varying cathode chemistries, and economic challenges hinder recycling efforts. Estimates show that a significant portion of Li-ion batteries from e-waste are recycled, but overall recycling rates remain low, raising environmental concerns associated with landfill disposal [8]. Several factors, including the uneven global distribution of lithium resources, fluctuating material costs, and environmental impacts of improper disposal, drive the urgency for effective EOL LIB recycling. LIBs contain a variety of valuable as well as potentially hazardous elements, including manganese, nickel, cobalt, lithium, and others [9]. Improper disposal of LiBs may lead to bioaccumulation and biomagnification of the hazardous elements in the aquatic and terrestrial food chain [10]. Whenever the casing is damaged and the internal components of a battery are exposed, the reaction between the electrolyte and water can produce toxic gases, including hydrogen fluoride (HF) [11], and may also result in fires or explosions [12]. Due to their potential risk for thermal runaway [13] and unpredictable behavior, lithium-ion batteries (LIBs) require thorough safety testing for transportation [3]. Establishing local recycling and repurposing centers for LIBs can mitigate risks, offer economic benefits, and maintain access to valuable materials within stricter regulatory frameworks [3].

Before deciding whether to repurpose a battery and send it to the end-of-life battery recycling process, determining the state of health (SOH) is an essential step in the process. LiBs are unsuitable for electric vehicles (EVs) when their power capacity falls below 80% and become unusable when their electric capacity drops below approximately 40% [14]. EOL EV batteries can be recycled, remanufactured, or used for another purpose depending on their remaining capacity, quality, chemistry, and state of health (SOH). In an ideal condition, recycling would come after remanufacturing or repurposing to maximize the value of LIBs [15]. One of the most environmentally friendly options is remanufacturing EOL batteries, which maximize value while reducing emissions and energy use. Repurposing EOL batteries for second-life uses, such as stationary storage, is an alternate approach. When remanufacturing is not economically feasible, this strategy maximizes the lifecycle value of the batteries by restructuring the battery system and updating the Battery Management System (BMS) for new uses, extending their usability beyond automotive applications.

**Figure 2:**
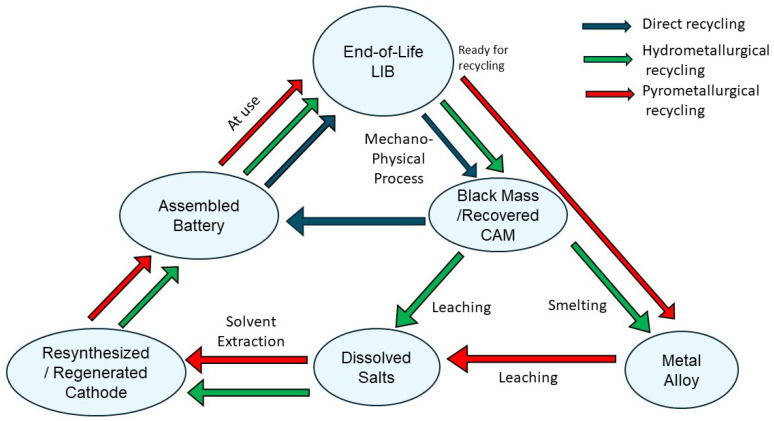
The blue, green, and red arrows indicate the three recycling methods. Mechano-physical processing (mechanical separation and dissolution) is usually adopted as a pretreatment for all these recycling routes (adapted with permission from [1]).

The batteries are first dismantled and crushed in the industrial-scale recycling process to obtain black mass [16]. The costs associated with breaking down LiBs are comparable to lead–acid batteries, primarily due to the use of similar equipment and similar operating expenses [17,18]. Black mass, a mixture of nickel, manganese, cobalt oxides, iron, and carbon, constitutes approximately 60% of the incoming EOL battery’s weight. In the three different recycling approaches—pyrometallurgical, hydrometallurgical, and direct recycling—the black mass is processed to extract valuable material that may be reutilized for battery production, as summarized in Figure 2.

Several review articles have emphasized the critical importance of efficient recycling processes for spent lithium-ion batteries (LIBs) to address environmental concerns and resource depletion. Lv et al. [11] reviewed various recycling processes, focusing on energy consumption, leaching, chemistry, and dissolution. Zhang et al. [19] highlighted strategies for recycling all LIB components, tackling the challenge of discarded batteries. Tagliaferri et al. [20] conducted a life cycle assessment of electric and hybrid vehicles, underscoring the environmental impacts of battery disposal. Winslow et al. [21] examined the growing concern over waste LIBs, emphasizing the need for effective management strategies. Paulino et al. [22] prioritized recovering essential elements, whereas Gastol et al. [23] stressed reusing and upcycling cathodes. To avoid landfill trash, Iturrondobeitia et al. [24] emphasized the significance of restoring end-of-life battery materials into the economic system. Huda et al. [25] stressed the importance of public knowledge and involvement in recycling programs while highlighting the necessity of appropriate waste battery disposal procedures. Ojanen et al. [26] called for unique design-for-recycling strategies and criticized using electrochemical discharge in salt solutions for recycling. The importance of battery recycling for sustainable waste management was highlighted by Rarotra et al. [27]. In their comparative life cycle assessment, Zhao and You [28] focused on the effects LIBs have on the environment, while Nigl et al. [29] evaluated the risk of fire in waste management systems and emphasized the importance of safety precautions. Furthermore, Zhao et al. [30] also highlighted the need for recycling by reviewing battery market patterns and second-life usage, while Krekeler et al. [31] raised policy issues by looking at the variety of used batteries. These studies highlight the need for improved recycling processes, safety protocols, and market-driven policies. However, given the research interest and importance of battery remanufacturing, there is a need to review the opportunities, challenges, and potential strategies for direct recycling.

Current industrial recycling processes for spent LIBs are thoroughly reviewed in this article, with special attention to developments in direct recycling techniques. We review the state of direct recycling technologies, including pretreatment techniques, regeneration procedures, and key material separation from wasted LIBs. Furthermore, the possibility of the direct recycling process to save energy consumption and enhance sustainability through material recovery, impurity elimination, and the creation of superior cathode materials is investigated. Finally, novel methodologies such as solvent extraction fractionation and cryo-mechanical treatment are investigated to emphasize their contribution to improving the sustainability and efficiency of industrial recycling procedures for lithium-ion batteries.

## 2. Battery Recycling

### 2.1. Pretreatment Process

**Figure 3:**
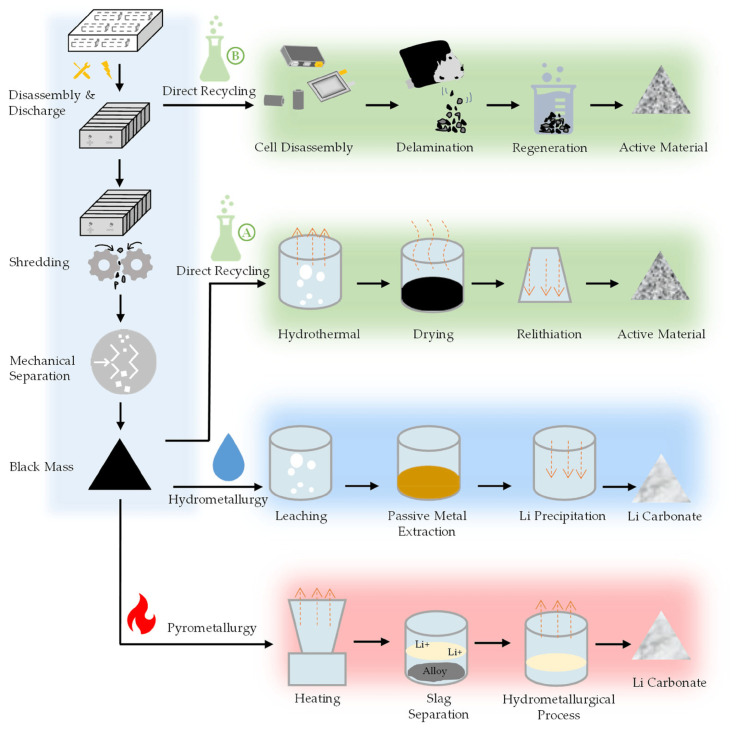
Typical recycling routes for spent LIBs (reproduced with permission from [37]).

The pretreatment process in battery recycling is essential for preparing the battery materials for further processing Ref. [32], including pyrometallurgical and hydrometallurgical methods [33], as shown in Figure 3. Physical pretreatment methods, such as deep discharge, crushing, physical separation processes, dissolution, leaching, and mechanical enrichment, are commonly applied to ensure efficient separation [11] and the recovery of valuable components from the batteries [34,35]. The pretreatment process is a crucial initial step in battery recycling, enabling the efficient recovery and reuse of valuable materials from spent lithium-ion batteries [36].

#### Pretreatment Steps

*Discharge and Dismantling*: Initially, spent batteries must be safely discharged to eliminate any residual charge, mitigating the risk of short circuits or thermal events during mechanical processing [33]. After that comes dismantling, in which batteries are mechanically or manually disassembled to extract the electronic components, wiring, and casing, leaving the core materials for processing. Researchers have reported automated or semiautomated approaches for this processing step. The first attempt at automation in disassembling was conducted by Gerber et al. [34].

*Shredding and Crushing*: The battery cells are further broken down into smaller pieces by shredding or crushing processes to facilitate the separation of the active components from the electrodes [37].

*Sieving and Sorting*: The split battery parts go through sieve procedures to separate the particles based on size. Air classification, gravity separation, and magnetic separation are used to separate materials based on their physical properties, such as density and magnetic susceptibility [32].

*Hydrometallurgical Conditioning*: Wet chemical pretreatment may be applied to the sorted fractions to leach out valuable metals, preparing them for hydrometallurgical recovery [38].

*Thermal Treatment*: Thermal methods, such as pyrolysis or calcination, may also be used during pretreatment to remove organic materials, such as binders and electrolytes, to enhance the purity of the metallic fractions and improve the efficiency of metal recovery [39].

### 2.2. Pyrometallurgical Process

**Figure 4:**
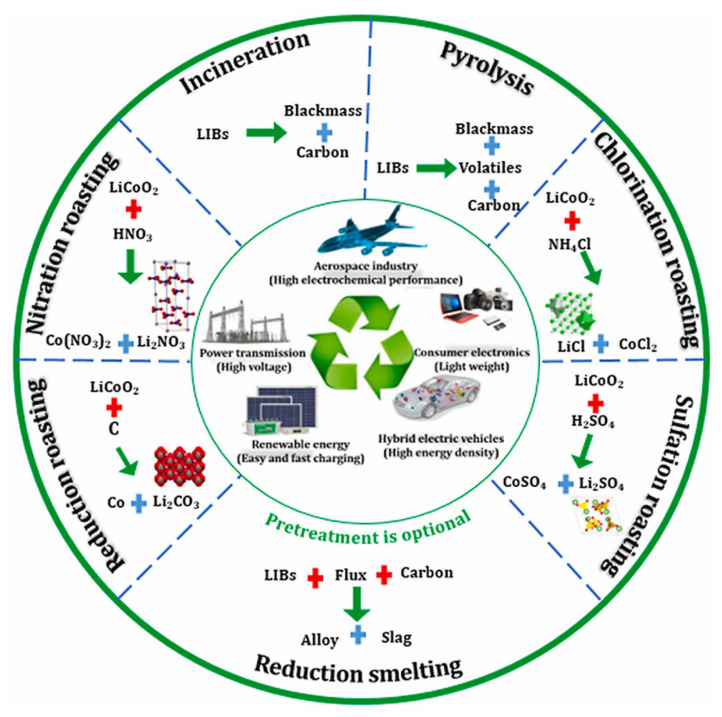
Different pyrometallurgical processing pathways (reproduced with permission from [41]).

The pyrometallurgical battery recycling method involves melting the battery components, often with added fluxes, to segregate metals into distinct phases. The resultant metal phase typically comprises cobalt, nickel, and some iron, while the slag phase contains lithium alongside other residual elements. As shown in Figure 4, subsequent steps are taken to extract and refine these materials for reuse [40]. Pyrometallurgical recycling of lithium batteries can be classified into two steps, and it is a well-established method for recovering NiMH and spent lithium-ion batteries [39].

*Smelting:* Smelting, the primary step in the pyrometallurgical process, involves raising the battery waste’s temperature in a furnace [41]. Fluxes like limestone or borax are usually added to lower the melting point, decrease viscosity, and facilitate the separation of metal from non-metallic components [42].

*Separation:* When the material melts, it divides into two primary layers: the heavier metal phase sinks to the bottom, while the lighter slag phase, which contains non-metallic components and certain metals such as lithium, floats on top [43]. This separation is critical for differential metal recovery [44].

*Recovery and Refining:* Metals in the molten state are then further refined to purify and qualify them for reuse in battery production or other applications [44]. The slag, which frequently contains important elements such as lithium, may require extra processing to recover these materials.

While effective in recovering metals like cobalt and nickel, the pyrometallurgical process is energy-intensive and can generate harmful emissions, necessitating robust environmental controls and efficient energy usage. The technique has various challenges, including the need for preliminary treatment processes to eliminate hazardous chemicals and maximize recovery efficiency. Current research focuses on upgrading furnace technologies, process controls, and slag management approaches to increase efficiency and environmental sustainability [45]. These developments are critical for overcoming the constraints of present methods and attaining more sustainable practices in the industry.

However, it is important to note that pyrometallurgical processes involve some disadvantages such as materials loss, hazardous gases release, dust emission, and high energy consumption [38]. Additionally, the operation of high-temperature pyrometallurgical processes almost always involves forming chemically aggressive liquid phases, such as slag and molten salts [46]. Furthermore, recent studies have demonstrated the importance of generating high lithium-containing phases by tailored slag composition and solidification to efficiently apply hydrometallurgical approaches to the pyrometallurgically gained slags, emphasizing the potential for optimization and efficiency [47].

### 2.3. Hydrometallurgical Process

**Figure 5:**
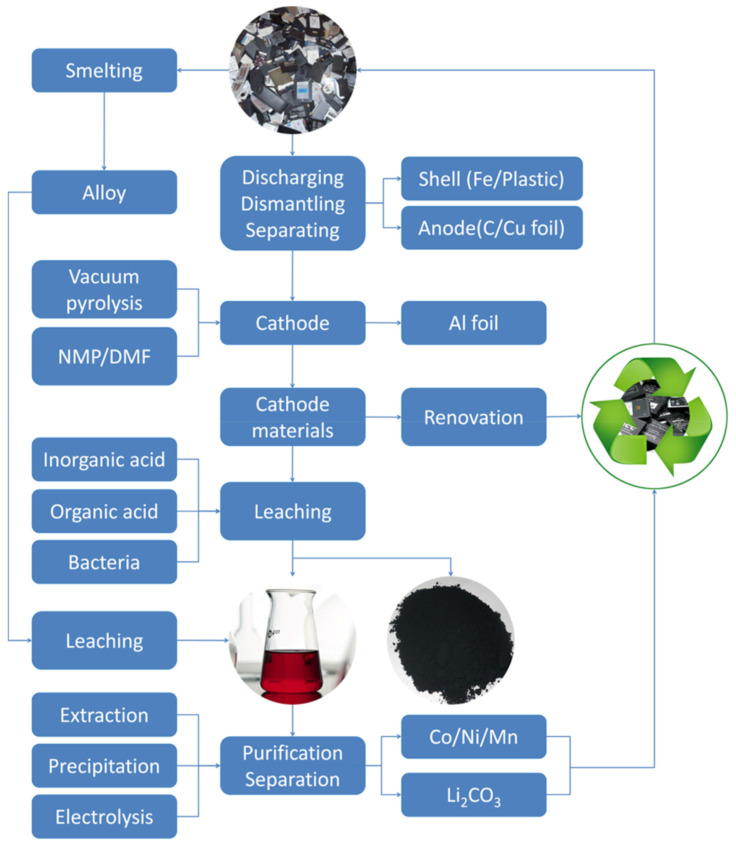
Typical hydrometallurgical recycling processing pathways of spent LIBs (reproduced with permission from [50]).

The hydrometallurgical process of battery recycling involves dissolving metals from battery components, which are further separated and purified to produce high-purity metal compounds, as summarized in Figure 5. Hydrometallurgical methods can achieve high-purity recycled metal compounds with a lower impact on health and the environment when compared to pyrometallurgical processes. Hydrometallurgy is frequently considered less hazardous and more environmentally friendly than pyrometallurgical processes [43]. It uses fewer resources and can be more discerning in selecting particular metals, processing at lower temperatures, and possibly lowering harmful emissions. The hydrometallurgical process has drawbacks despite its benefits, such as the requirement for huge amounts of water and chemicals, which can cause problems with waste management. Lithium-ion batteries are inherently unstable. During recycling, if the barrier between the cathode and anode ruptures, it can trigger a thermal reaction, leading to high temperatures, ignition, or explosion [48,49]. Lithium batteries contain hazardous materials. The electrolytes in the batteries can cause fires, and there is a risk of toxic dust explosions. Throughout the recycling process, doped elements may leach out or coating layers may be destroyed, affecting the quality of regenerated cathode materials. Enhancing the hydrometallurgical recycling processes’ economy, sustainability, and efficiency is the main goal of ongoing research. Active research is being performed in the areas of leaching agent innovation, more effective separation methods, and closed-loop systems that reduce waste and environmental effects.

#### 2.3.1. Hydrometallurgical Process Steps

*Leaching*: Leaching is the process of utilizing a chemical solution called a lixiviant or leaching agent to extract precious metals from the cathode components of discharged lithium-ion batteries. Because it converts the metals in the solid battery components into a soluble state that can be treated in the following stages, this step is crucial to the recovery of metals. The choice of leaching solution is influenced by factors such as the types of metals to be recovered, the composition of the cathode, environmental regulations, and the desired purity of the product. The initial stage involves chemically breaking down battery components in a solution to release metals in soluble form. Typically, leaching media, such as inorganic acid, organic acid, alkali, or bacterial solutions, are used to extract cathode active components. Occasionally, additional techniques like mechanical chemistry and ultrasonic waves are used to improve the leaching process. The most widely utilized leaching agents for the leaching of cathode active materials are a number of inorganic acids, including H_2_SO_4_ [51,52], HCl [52,53], and HNO_3_ [54]. Certain organic acids exhibit favorable characteristics when compared to inorganic acids, such as quick degradation, recyclable nature, low likelihood of secondary environmental damage, and sufficient acidity to dissolve the cathode active components [35,55,56]. Bioleaching is an appealing substitute for inorganic and organic acid leaching due to its less demanding industrial application, reduced cost, and ecological benefits [57,58]. The interaction of hydrogen ions and cathode active materials in an acidic solution is the fundamental process of inorganic acid leaching, organic acid leaching, and bioleaching. The interaction of metals with hydroxide ions in an alkaline environment is known as alkaline leaching. NaOH and ammonia are widely used alkaline leaching reagents [59,60]. Occasionally, additional techniques like mechanical chemistry and ultrasonic waves are used to improve the leaching process [61].

*Separation*: To separate specific metals from the leachate, specific procedures are used, such as solvent extraction, ion exchange, or precipitation. To achieve high purity levels in the recovered metals, this process is essential.

*Purification and Recovery:* To get rid of any remnant impurities, the separated metals undergo additional purification. Metal ions in solution can be converted into solid metal forms or high-purity chemical compounds by processes like chemical precipitation or electrowinning, which are then available for use in battery production or other applications.

**Figure 6:**
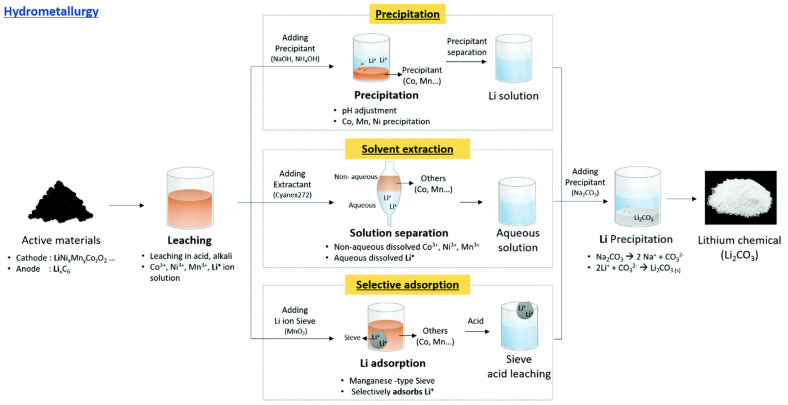
Overall schematic of lithium recycling from pre-treated waste LIB components by hydrometallurgy process (reproduced with permission from [62]).

The synthesis of ready-to-use precursors for cathode active materials in hydrometallurgy can be accomplished through the series of steps depicted in Figure 6. Following the leaching phase, which separates active components into a solution comprising metal ions such as Co^2+^, Ni^2+^, Mn^2+^, and Li^+^, the ensuing processes include precipitation, solvent extraction, and selective adsorption. These methods are systematically utilized to separate and purify lithium ions, ultimately yielding lithium carbonate (Li_2_CO_3_), an essential precursor for the manufacture of cathode materials.

#### 2.3.2. Direct Recycling Process

The direct recycling technique for batteries involves retrieving and repurposing individual components from used batteries without significantly changing their form or structure [63]. By completing the cycle and reducing the demand for additional resources, this technique aims to incorporate the recovered elements into new batteries. Direct recycling processes preserve the integrity of the original materials, especially the cathode and anode components of lithium-ion batteries, and encourage a circular economy by reducing the environmental impact of battery manufacture and disposal. Even with the continued difficulties in material synthesis and separation, research and development efforts are concentrated on improving direct recycling methods to raise the effectiveness and sustainability of battery recycling procedures.

## 3. State-of-the-Art Direct Recycling Process

The technique of recovering, renewing, and repurposing battery components without altering their chemical structure is known as direct recycling. It has also been referred to as cathode-to-cathode and direct cathode recycling. The paradigm of battery recycling has drastically changed with the introduction of direct recycling, which emphasizes sustainability and conservation. It is possible to bypass several expensive and energy-intensive processing steps by directly regenerating cathode active material. Direct recycling reduces the demand for limited resources by reusing these materials while supporting a more equal and equitable distribution of these vital minerals. Furthermore, by prolonging the life cycle of battery components, direct recycling contributes to waste reduction, another vital aspect of environmental preservation.

**Figure 7:**
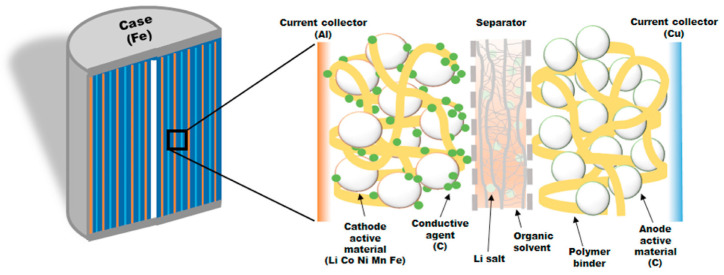
A schematic diagram for typical lithium battery components (reproduced with permission from [64]).

Direct Recycling Process Steps: Figure 7 shows the typical battery components and the materials that need to be separately treated in case of direct recycling:

1. Cathode Materials: Lithium metal oxides like LiCoO_2_ (LCO), LiNi_x_Co_y_Mn_z_O_2_ (NCM), LiMn_2_O_4_ (LMO), and LiFePO_4_ (LFP). These are common cathode materials and contain valuable elements for recycling.
2. Anode Materials: Graphite, Si, TiO_2_, and alloys are commonly used as anode materials. These can be partially reused.
3. Electrolyte: The electrolyte consists of lithium salts (such as LiPF_6_, LiBF_4_, and LiClO_4_) combined with organic solvents (e.g., propylene carbonate, ethylene carbonate, ethyl methyl carbonate, dimethyl carbonate, and diethyl carbonate). These form the conductive pathway for lithium ions to move.
4. Other Components:
  - Separator: Typically made of polyethylene or polypropylene, it serves as a barrier between the cathode and anode.
  - Plastic Shell: The outer casing of the battery, which is considered waste.

Current Collectors: Aluminum (Al) and copper (Cu) current collectors can be recycled.

To prevent any short circuits or heat events while handling further down the line, the batteries need to be completely discharged, and for large-scale applications, conductive chemical solutions like NaCl, FeSO_4_, and MnSO_4_ are excellent discharging mediums [65]. In direct recycling, electrochemical discharge methods are commonly used [33]. After being completely drained, the batteries are carefully disassembled, a procedure that can be completed manually or by automated systems.

### 3.1. Harvesting Cathode and Anode Materials

The initial phase involves recovering cathode and anode materials separately from spent batteries. If battery cells are crushed, an efficient separation process is necessary to isolate the metal-containing components from other materials, such as polymers (from the separator) and battery casings. The cathode and anode materials need to be separated from the current collectors. The process of separating the cathode active material from the aluminum foil and the anode materials from the copper foil is an important area of interest for researchers.

### 3.2. Cathode Active Material (CAM) Separation

Typically, methods that dissolve the aluminum (Al) foil or remove the polyvinylidene fluoride (PVDF) binder are employed to separate CAMs from aluminum. The PVDF binder can be separated by dissolution or decomposition. Al can be separated as pieces via delamination or dissolution [66]. For dissolution, Hansen solubility parameters were investigated along with the solubility behaviors of PVDF in 46 solvents [67]. Several solvents were tested for their capacity to separate cathode components while recycling lithium-ion batteries (LIBs); N-N-dimethylformamide (DMF), N-N-dimethylacetamide (DMAC), N-N-dimethyl sulfoxide (DMSO), and ethanol were among the solvents that were investigated [68,69]. The results showed that NMP was the most efficient solvent, with DMAC, DMF, DMSO, and ethanol following, in that order. Using ultrasound technology promoted the solvent’s convective motion and produced ultrasonic cavitation to improve the process’s efficiency. It was determined that 240 watts of ultrasonic power, a 70 °C temperature, and a 90 min reaction time were the ideal parameters for this operation. Other sustainable procedures to reduce the environmental impact connected to separate CAMs are discussed in literature [70].

#### 3.2.1. Deep Eutectic Solvents (DESs) for Delamination

Choline chloride and glycerol were used to delaminate NMC111 cathode materials by weakening the bonds between the cathode and the Al foil through hydrogen bond formation. The process operates at 190 °C and achieves a peel-off efficiency of 99.87% within 15 min [71].

#### 3.2.2. Ethylene Glycol Method (Fast Delamination)

Ethylene glycol can peel off NMC523 cathode material in 6 s with a solid-to-liquid ratio of 1:10 at 160 °C. The effectiveness of ethylene glycol is attributed to the strong hydrogen bonds it forms with the oxidation layer of the Al foil, which replaces the weaker bonds between PVDF and the foil. This rapid action is independent of the size of the cathode pieces due to the solvent’s diffusion and the porous nature of the cathode [72].

#### 3.2.3. Fatty Acid Methyl Esters (FAMEs)

This method is derived from waste oil and utilizes waste oil, methanol, and NaOH to produce FAMEs, which then dissolve PVDF at 190 °C. It achieves a 99.1% peel-off efficiency within 20 min. This approach reduces environmental impacts since the reagents are derived from waste materials [73].

#### 3.2.4. Physical Separation

Physical separation is a low-temperature method specifically for LCO cathodes that involves deactivating PVDF by cooling it to low temperatures (77 K), followed by grinding. It recycles 87.29% of the LCO cathode material [74].

#### 3.2.5. Molten Salt Treatment (AlCl_3_-NaCl System)

Molten salt treatment is performed at 160 °C with a duration of 20 min and an AlCl_3_-NaCl molar ratio of 1:1. This approach attains a peel-off efficiency of 99.8%, functioning as both a heat transmission medium and a melting agent for PVDF [75].

#### 3.2.6. Binary Eutectic System

A binary eutectic system of LiOH and LiNO_3_ breaks bonds among cathode active material (CAM) particles at 260 °C, directly converting the cathode layer into powder and eliminating mechanical separation [76].

#### 3.2.7. Thermal Decomposition

Cathode scraps or sheets are calcined at 500–600 °C [77,78]. The solid-state synthesis [79] involves a recycling apparatus to ensure a thermo-mechanical recycling process.

#### 3.2.8. Flotation

In battery recycling, flotation is a technique that separates cathode active materials (CAMs) from carbon and other materials, like PVDF, while producing no hazardous emissions, such as HF. To improve the separation based on hydrophobic and hydrophilic traits, the materials are typically crushed, screened, and treated with collectors such as methyl isobutyl carbinol and foaming agents like n-dodecane. For example, the hydrophobicity of graphite is enhanced to distinguish it from CAMs, which is rendered hydrophilic. This leads to notable percentages of graphite and CAMs being separated in their respective flotation stages, resulting in excellent recovery rates. Although they have a lower recovery rate, techniques like Fenton reaction-assisted flotation further enhance the process by eliminating organic layers and increasing the effectiveness of separating components like LCO (CAM) and graphite. In addition to efficiently separating the components, this technique reduces the amount of impurities like carbon black and PVDF and contributes to recycling a substantial portion of CAMs [80,81].

### 3.3. Separation of Anode Materials from Binders and Conductive Agents

Since most binders, like SBR, used on the anode side are soluble in water, adding water makes it easy for materials to separate from the copper current collector.

### 3.4. Separation of Metal-Containing Components from Polymers

Separating metal-containing components from polymers in batteries is essential for recycling and recovering valuable materials. Batteries, particularly lithium-ion types, consist of metals like lithium, cobalt, and nickel, as well as various polymers as binding agents in electrolytes and casings. The separation process often begins with mechanical techniques such as shredding and sieving to reduce size and differentiate components based on density. Chemical methods, including dissolution and acid leaching, can selectively remove polymers while leaving metals intact. Thermal treatments like pyrolysis decompose polymers in an oxygen-free environment, while incineration can eliminate them through combustion, albeit with emission concerns. Electrochemical methods also allow for selective metal recovery from solutions. After separation, the extracted metals are refined for purity, and any remaining polymers can be reprocessed for reuse. Overall, these methods aim to maximize recovery rates and minimize environmental impacts, contributing to more sustainable battery recycling practices.

### 3.5. Regeneration

Recovered metals can be converted back into metal oxides, which can be used as raw materials for new battery cathodes. Direct recycling methods for lithium-ion batteries (LIBs) focus on restoring the electrochemical performance of spent cathode materials (CAMs), which often suffer from capacity loss due to various degradation mechanisms. Researchers have pinpointed active lithium loss as a primary factor contributing to this degradation. This loss can be attributed to the deterioration of the battery’s surface during use, reactions with impurities on the surface, and the formation of a solid electrolyte interphase (SEI) layer.

To address these issues and regenerate CAMs, a few re-lithiation approaches are usually incorporated:

- Solid-state sintering;
- Hydrothermal re-lithiation;
- Ionic liquid method;
- Molten salt approach;
- Electrochemical re-lithiation;
- Some other techniques that depend on cathode chemistry.

#### 3.5.1. Solid-State Sintering

Solid-state lithium reagents are used as sources of lithium in solid-state re-lithiation processes. Lithium ions are incorporated into accessible sites in a material at high temperatures in this technique, which effectively compensates for the lithium loss during battery charge and discharge cycles. Lithium hydroxide, nitrate, and carbonate are common sources of lithium used during this method. These substances supply the lithium ions required to restore and preserve the battery’s capacity and functionality during prolonged operation. The lithium ions are reinserted into the material’s crystal structure through the application of heat, guaranteeing the battery’s durability and effectiveness. Lithium carbonate is the most commonly used source of lithium for solid-state sintering re-lithiation, especially for various battery chemistries. This process typically occurs at elevated temperatures of around 850 to 900 degrees Celsius [82,83]. At these high temperatures, lithium carbonate provides a stable and effective supply of lithium ions, which are incorporated into the material to replenish the lithium lost during charge–discharge cycles.

**Figure 8:**
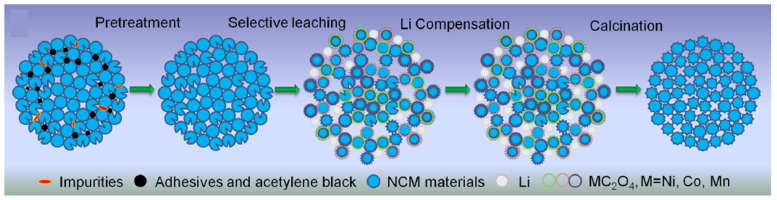
The regeneration mechanism of CAM by selective leaching combined with the calcination process (reproduced with permission from [85]).

Lithium-ion diffusion is accelerated by integrating mechanical activation with the solid-state process. A mixture of Li_2_CO_3_ and NMC (nickel manganese cobalt) is sintered for ten hours at 800 °C after mechanical activation. By lowering the amount of nickel and catanionic disorder, this method of combining mechanical activation with sintering improves NMC’s discharge capability and recovers its layered structure. As a result, the performance and efficiency of NMC material in battery applications are greatly enhanced by this technique [84]. The selective leaching method with calcination is one of the effective methods that regenerate the crystal structure. Figure 8 illustrates the incorporation of selective leaching with oxalic acid and solid-state calcination of metal oxalates, which substantially facilitates the resynthesis of cathode active materials.

#### 3.5.2. Hydrothermal Re-Lithiation

**Figure 9:**
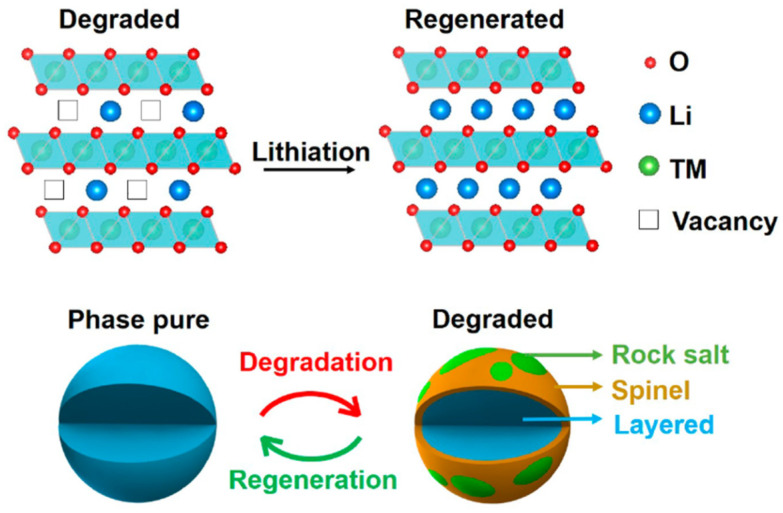
Illustration of the hydrothermal lithiation process in which Li^+^ is re-dosed to Li-deficient sites to recover its desired stoichiometry (reproduced with permission from [86]).

Lithium hydroxide solution is used as a common source of lithium in hydrothermal re-lithiation. Hydrothermal conditions, which usually entail high pressure and temperature within an aqueous solution, are employed in this procedure to incorporate the lithium ions into the material. To stabilize the structure and guarantee that the lithium ions are properly integrated into the material’s lattice, an annealing step is often carried out at a temperature of approximately 800 °C after the hydrothermal treatment [82]. As shown in Figure 9, Shi et al. also developed an efficient method that involves hydrothermal re-lithiation using a 4 M LiOH solution, followed by either a solid-state sintering step or a short annealing process [86].

To improve the transfer and diffusion of lithium ions during hydrothermal re-lithiation, a number of cutting-edge methods are employed. One such method is ultrasonication, in which high-frequency sound waves form small cracks in the mixture to improve mixing and increase the area of contact between the material and the lithium source. As a result, lithium ions are distributed more evenly and efficiently [87].

A different method that is employed is called aqueous pulsed discharge plasma, and it produces short, intense electrical pulses in the aqueous solution. These pulses create plasma, a very reactive state of matter that speeds up the diffusion of lithium ions into the material and allows for quick chemical reactions. By optimizing the re-lithiation process, this technique raises the general efficacy and efficiency of lithium incorporation [88].

**Figure 10:**
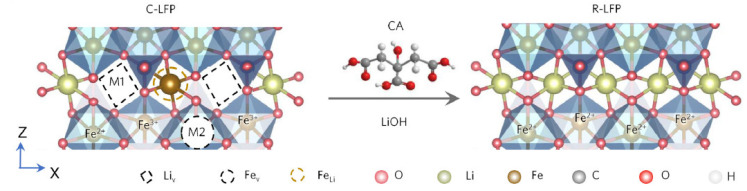
Schematic illustration of the solution re-lithiation process. The positions of Li and Fe in a perfect olivine structure are defined as sites M1 and M2, respectively. The left side shows C-LFP with Li vacancies and Fe occupation in a Li site (FeLi); the right side shows R-LFP with all the Fe^3+^ being reduced to Fe^2+^ with the presence of CA in a LiOH solution (reproduced with permission from [89]).

The solution re-lithiation procedure, which involves the presence of a critical acid in a LiOH solution, is a promising technique that is particularly effective for LFP batteries. (Figure 10).

#### 3.5.3. Ionic Liquid Method

The ionic liquid re-lithiation method is an innovative approach to battery recycling and re-lithiation, offering several advantages over traditional methods [90]. This process uses ionic liquids as a medium to facilitate the re-incorporation of lithium ions into spent cathode materials. Ionic liquids are salts that are liquid at or near room temperature and possess unique properties such as low volatility, high thermal stability, and excellent solubility for a wide range of compounds [91]. These characteristics make ionic liquids particularly suitable for high-temperature, solid-state reactions. A class of substances known as ionic liquids (ILs) are generally described as salts made entirely of ions, usually consisting of organic cations combined with either inorganic or organic anions. In contrast to traditional salts, ionic liquids maintain a liquid form at comparatively low temperatures, frequently below 100 °C. Organic cations typically encompass imidazolium, pyridinium, ammonium, or phosphonium groups, whilst the anions may vary from halides and nitrates to more intricate species such as bis(trifluoromethylsulfonyl)imide [92]. Various lithium sources have been tested for this process, including lithium acetate (LiOAc), lithium bis(trifluoromethanesulfonyl)imide (LiNTf), lithium bromide (LiBr), and lithium chloride (LiCl). Among these, LiBr has proven to be the most effective. This superiority is attributed to the fact that bromide ions (Br^−^) are easier to oxidize (Br^−^/Br_2_ at 1.065 V). The oxidation process forms bromine (Br_2_), which is then removed from the system, thereby facilitating the lithiation reaction.

Spent NMC materials can be mixed with the preselected lithium source and the ionic liquid. This mixture can then be heated to around 150 °C for approximately 6 h [91]. The temperature and duration can be adjusted depending on the specific materials and desired outcomes. At elevated temperatures, the ionic liquid acts as a flux medium, promoting the diffusion of lithium ions into the NMC structure. The presence of the ionic liquid enhances the mobility of lithium ions, facilitating their incorporation into vacant sites within the NMC lattice. Usually, after the initial re-lithiation, the material often undergoes an annealing process at higher temperatures (around 800 °C) to stabilize the structure and enhance the electrochemical performance.

The large-scale application requires careful consideration of cost, particularly the cost of ionic liquids, which are a significant expense in the process. Impressively, 98.9% of the ionic liquid can be recovered and recycled in these large-scale operations, making the process more cost-effective and sustainable [93]. This efficient recycling of ionic liquid helps maintain the economic and environmental feasibility of the process [94,95], ensuring that the benefits of using ionic liquids are maximized.

The ionic liquid re-lithiation method represents a significant advancement in battery recycling technology. By leveraging the unique properties of ionic liquids, this method offers a highly effective, environmentally friendly [96], and scalable solution for restoring spent cathode materials. The ability to recycle ionic liquids further enhances the sustainability and economic viability of this process, making it a promising approach for the future of battery recycling and re-lithiation.

#### 3.5.4. Molten Salt Approach

The molten salt method of direct recycling uses liquified lithium salts to insert Li-ions into spent cathodes, regenerating lithium with other valuable materials for the battery’s cathode. It has been found that combining certain lithium salts together can reduce the melting point, therefore minimizing the energy needed for the extraction. These mixed salts are called eutectic salts, and by testing different variations, the efficiency of the molten salt method can be optimized.

Based on the application of lithium hydroxide (LiOH) in hydrothermal processes, scientists used a simple molten salt system consisting of LiOH and potassium hydroxide (KOH). With this combination, a eutectic mixture is created that has a lower melting point than any of its constituent parts. Because lithium carbonate (Li_2_CO_3_) is more reactive than lithium hydroxide (LiOH), it can also be chosen as an additional source of lithium.

**Figure 11:**
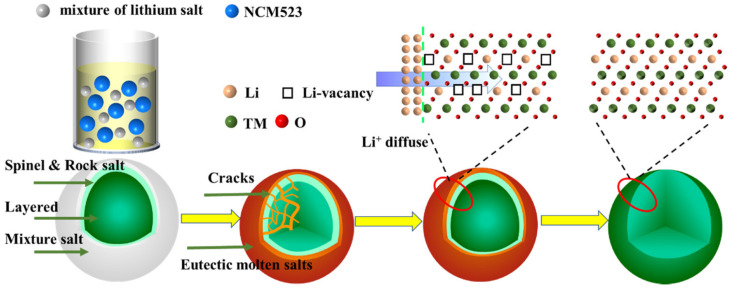
The re-lithiation process for Li composition recovery by the eutectic molten salt approach (reproduced with permission from [97]).

Jiang et al. demonstrated a successful strategy for regenerating NMC cathode materials using a common eutectic salt mixture of LiOH and Li_2_CO_3_, combined with a two-step calcination process. This approach facilitates the incorporation of excess lithium into the crystal structure, effectively restoring the cathode’s integrity. The process is illustrated in Figure 11, showcasing the structural evolution and lithium diffusion during regeneration [97].

**Figure 12:**
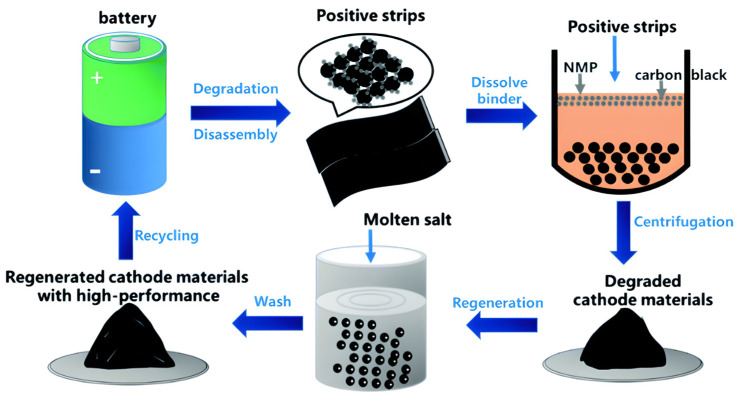
Illustration of the recycling and regeneration procedure (molten salt approach, reproduced with permission from [98]).

**Figure 13:**
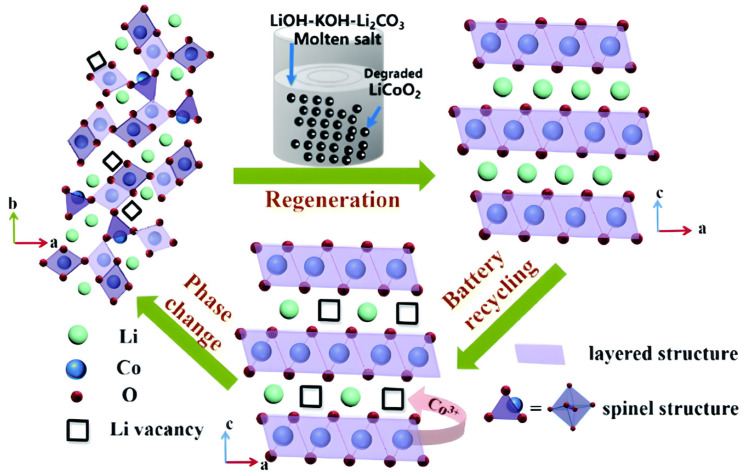
Illustration of the failure and regeneration processes of LiCoO_2_ about its Li composition and structural change (reproduced with permission from [98]).

As shown in Figure 12, Yang et al. successfully demonstrated a novel, nondestructive approach for regenerating degraded LiCoO_2_, enabling its direct reuse as a cathode active material in a LiOH–KOH–Li_2_CO_3_ molten salt system. This method leverages the molten salt’s high concentration of lithium ions and its role as a “solvent”, which creates a robust oxidative and dissolutive environment to effectively remove carbon, the binder, and other impurities. Under optimized conditions at 500 °C, the spent LiCoO_2_ cathode was regenerated to a quality comparable to commercial LiCoO_2_. The selection of the LiOH-KOH-Li_2_CO_3_ eutectic molten salt system was driven by its demonstrated efficiency in directly restoring damaged lithium cobalt oxide (LiCoO_2_) through a simple one-pot thermochemical treatment, as illustrated in Figure 13 [98].

**Figure 14:**
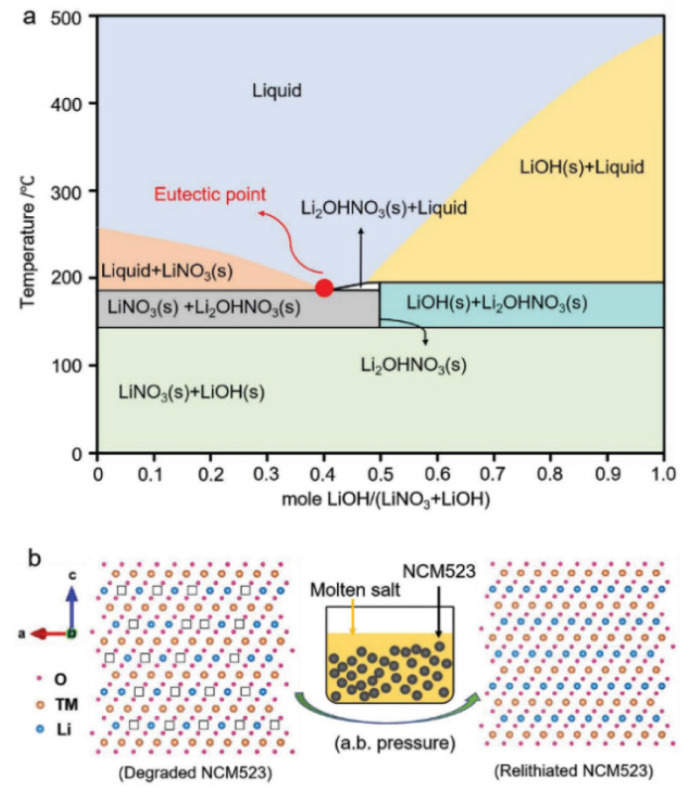
(**a**) Phase diagram of eutectic salts. (**b**) Visual depiction of the transition from spent cathode to regenerated cathode using the molten salt approach (reproduced with permission from [99]).

Shi et al. were the first to demonstrate the ambient-pressure re-lithiation of degraded LiNi_0.5_Co_0.2_Mn_0.3_O_2_ (NCM523) cathodes using eutectic Li^+^ molten salt solutions composed of LiOH and LiNO_3_. Through a low-temperature re-lithiation process at 300 °C, combined with subsequent thermal annealing, the degraded NCM523 cathodes were effectively restored to their original composition, crystal structure, and electrochemical performance under ambient pressure conditions [99]. The phase diagram of the eutectic salts, along with a visual representation of the transition from spent cathode to regenerated cathode using the molten salt approach, is presented in Figure 14.

The primary advantages of the molten salt method are that it causes minimal damage to the physical structure of the cathode, is economically efficient and environmentally friendly, and has a high retention rate of lithium ions. Additionally, molten salts have high reactivity, high volatility, and great solubility. Outweighed greatly by its advantages, the disadvantages of the molten salt method are that it requires more materials than some of the other methods and requires a heating process [100]. Although, the heating is not extensive, especially when more effective eutectic salts are used.

#### 3.5.5. Electrochemical Re-Lithiation

An efficient approach for the rejuvenation and segregation of cathode active material entails the application of electrochemical methods. This method facilitates use within electrochemistry to efficiently regenerate deteriorated cathodes while minimizing waste, as it requires no additional chemical reagents. Electrochemical insertion and electrodeposition are important methods in this field. These techniques make it easier to separate and recover specific ions from intricate configurations, resulting in them being ideal for purposes such as reclaiming lithium from expired batteries and industrial waste sources [101,102].

**Figure 15:**
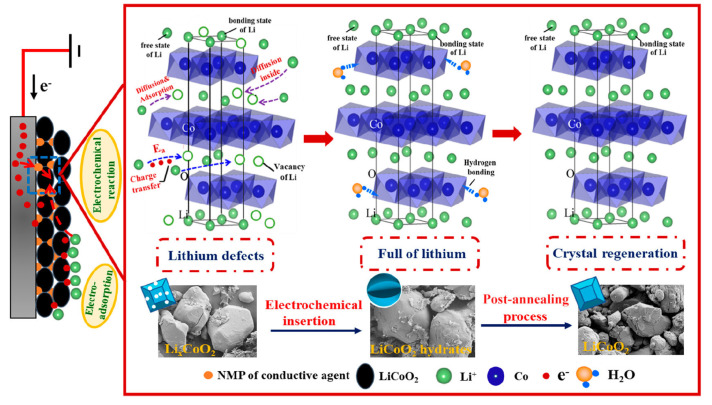
The mechanism of direct regeneration of LCO by electrochemical lithiation insertion (reproduced with permission from [104]).

Electrodeposition involves the transfer of reducing ions from a solution to a conductive substrate in order to form a solid layer of material. This technique is frequently used to enhance the durability and corrosion resistance of surfaces by applying copper, nickel, and chromium deposits. Ions are inserted into a host material’s lattice structure through a process called electrochemical insertion, sometimes referred to as intercalation, without seriously disrupting the host’s structure. This method serves very well when producing battery electrode materials. One useful cathode material for lithium-ion batteries is lithium manganese dioxide (LiMnO_2_), which is produced when lithium ions intercalate into manganese dioxide (MnO_2_) structures [103]. Zhang et al. [104] developed a process for electrochemical re-lithiation, as depicted in Figure 15. Electrons from a power source are carried through a conductive medium to the cathode, facilitating the migration of Li^+^ ions into lithium vacancies within the Li_x_CoO_2_ framework. When the electrode’s energy surpasses the activation energy (Ea), Li^+^ ions exceed the energy barrier and integrate into the defective lattice. Certain Li^+^ ions may occupy interstitial locations within the layered structure, establishing hydrogen bonds that induce particle expansion due to crystalline water. An annealing technique at 700 °C is employed to eliminate crystalline water and reinstate the lattice structure.

Li^+^ can be selectively inserted to form LiFePO_4_ in the two-phase system of LiFePO_4_/FePO_4_ by entropy mixing and the desalination technique. This happens after quick charging and discharging with a fixed potential [105]. Typically, Li^+^ ions are incorporated into the crystal structure to regenerate LiCoO_2_ for practical applications, and the recovery of Li^+^ in the structure by electrochemical methods is more viable [104].

#### 3.5.6. Other Innovative Techniques

**Figure 16:**
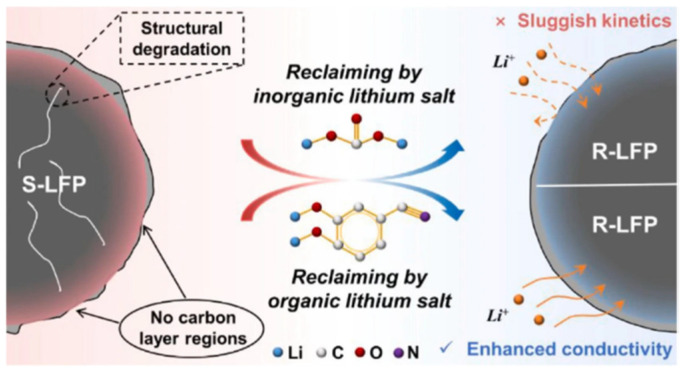
Schematic diagram of the mechanism of direct regeneration of LFP using organic salt (reproduced with permission from [106]).

**Figure 17:**
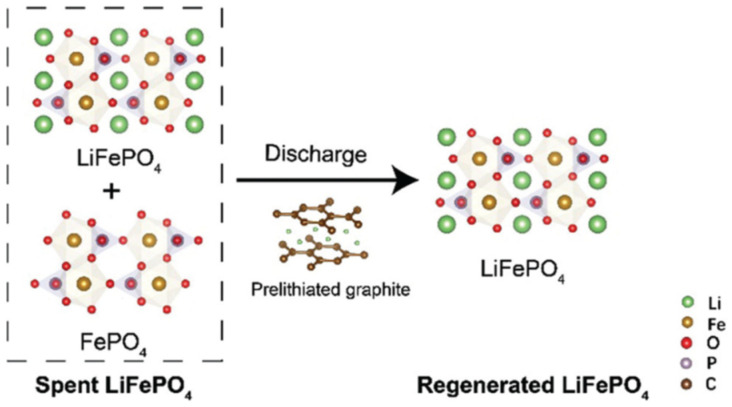
Schematic diagram of crystal structure evolution from FePO_4_ to LFP (reproduced with permission from [107]).

LiFePO_4_ batteries, commonly used in electric vehicles and energy storage systems, degrade over time due to lithium loss and the formation of Fe(III), which causes capacity fade. Additionally, the poor electrical conductivity of LiFePO_4_ limits its performance. A new approach uses an organic lithium salt, 3,4-dihydroxybenzonitrile di-lithium, to directly regenerate degraded LiFePO_4_ cathodes. This method, as shown in Figure 16, restores lithium vacancies and inhibits the formation of Fe(III) through the creation of a reductive atmosphere from the cyano groups in the salt. During the regeneration process, the salt pyrolyzes, forming a conductive carbon layer on the LiFePO_4_ particles, which improves both Li-ion and electron transfer. This restored cathode shows enhanced cycling stability and a high-capacity retention of 88% after 400 cycles at 5 °C. The technique also holds the potential for regenerating other transition metal oxide-based cathodes [106]. As illustrated in Figure 17, another study showed a direct regeneration method of LFP cathode using a graphite pre-lithiation strategy [107].

Karati et al. proposed an environmentally viable recycling method for large-capacity nickel manganese cobalt (NMC) batteries, utilizing electrochemical lithium concentration on the anode, followed by recovery using only water [108]. This method facilitates the effective recovery of all pouch cell constituents, encompassing lithium, graphite, copper, and aluminum current collectors, the separator, and the cathode active material. The delithiated cathode active materials resulting from this procedure can subsequently be restored using any of the current regeneration methods.

#### 3.5.7. Anode Material Regeneration

Most binders used in anodes are water-soluble, allowing for the efficient separation of graphite from the copper current collector using water [108]. Graphitic anodes are susceptible to various failure mechanisms, including solid electrolyte interphase (SEI) decomposition, dendrite formation, graphite cracking and exfoliation, and copper corrosion [109]. Finding appropriate regeneration processes for graphite depends critically on the degree of anode degradation, which is assessed by the cycle life. Dong et al. proposed a fast, efficient, and environmentally friendly method for regenerating graphite in end-of-life lithium-ion batteries using the Flash Joule Heating (FJH) technique, achieving excellent electrochemical properties [110]. Another study demonstrated the successful separation of copper foil and graphite from the anode materials of spent lithium-ion batteries using an electrolysis method. The recovered graphite was subsequently reused to prepare new anode materials, which exhibited excellent electrochemical performance [111]. Natarajan et al. demonstrated the synthesis of reduced graphene oxide (rGO) using metallic casings (Al and stainless steel) as reducing agents in the presence of concentrated HCl. The resulting rGO can be utilized as a supercapacitor material or further processed for reuse in anodes [112]. The presence of residual impurities in directly recycled anodes results in a reduced graphite content, which consequently leads to a lower specific capacity [113]. Pyrolysis treatment is a widely used method for removing impurities prior to regenerating graphite anodes (GAs) from spent lithium-ion batteries (LIBs). This process effectively eliminates electrolytes and binders, facilitating subsequent regeneration steps [109]. Yang et al. proposed an acid-leaching method for regenerating high-purity graphite anodes (GAs) following a two-stage calcination process [92]. To further improve the purity of the regenerated graphite, a combined approach integrating sulfuric acid curing, leaching, and calcination has also been suggested [114].

## 4. Conclusions

Traditional recycling methods may contribute to environmental degradation by producing hazardous byproducts or emitting contaminants into the air and water. Direct recycling is a more environmentally responsible alternative because it reduces those negative impacts. Although direct recycling may necessitate an initial investment in specialized equipment and processes, it can be cost-effective in the long term. This process repurposes valuable materials and can reduce the overall production costs of batteries by reducing the need for expensive raw materials. In addition, its cost-effectiveness is further enhanced by the potential savings from diminished energy consumption and waste management expenses.

One of the key advantages of direct recycling is its ability to retain the integrity of the original materials, particularly the cathode components. Direct recycling ensures that the recovered materials maintain their electrochemical properties and performance characteristics by avoiding extensive chemical breakdown or structural alterations. This preservation of material integrity enhances the quality and reliability of recycled batteries, making them suitable for various applications. Direct recycling promotes a sustainable and closed-loop approach to resource management by reintegrating recovered materials into new battery production. This integration reduces waste generation, promotes resource efficiency, and fosters a more sustainable model of production and consumption. By preserving the crystal structure of the cathode material, direct recycling minimizes the generation of waste and pollutants typically associated with battery recycling processes. Hence, Direct recycling presents a viable approach to address the increasing need for energy utilization while mitigating the environmental consequences of battery disposal and reproduction.
